# How to identify juxtaglomerular cell tumor by ultrasound: a case series and review of the literature

**DOI:** 10.1186/s12880-024-01220-9

**Published:** 2024-02-16

**Authors:** Li Wang, Meiying Li, Siqi Jin, Yunshu Ouyang, Fenglan Wang, Ke Lv, Jianchu Li, Yuxin Jiang, He Liu, Qingli Zhu

**Affiliations:** 1 Department of Ultrasound, Peking Union Medical College Hospital, Chinese Academy of Medical Sciences, Shuaifuyuan 1, Dongcheng District, 100730 Beijing, China; 2 Department of Ultrasound, Tangshan Central Hospital, West of Youyi Road, Lubei District, 063000 Tangshan City, Hebei China

**Keywords:** Juxtaglomerular cell tumor, Ultrasound, Diagnosis, Imaging

## Abstract

**Purpose:**

To study the value of ultrasound in the diagnosis of juxtaglomerular cell tumor (JGCT).

**Methods:**

From January 2005 to July 2020, fifteen patients diagnosed as JGCT by surgical pathology in Peking Union Medical College Hospital were collected. All patients underwent preoperative ultrasound examination. The clinical, laboratory, ultrasound, computed tomography (CT), surgical, and pathological features of the patients were analyzed retrospectively.

**Results:**

The 15 patients were 5 males and 10 females with a median age of 29 years (10∼72 years). 14 of them had hypertension and one had normal blood pressure. The tumors were all solitary, with a median diameter of 1.5 cm (0.9–5.9 cm). Among the fifteen patients, eleven were correctly detected by preoperative ultrasound, and four were missed. There was a significant difference in tumor size (2.64 ± 1.48 cm vs. 1.23 ± 0.21 cm) and whether the tumor protruded outward (9/11 vs. 0/4) between the ultrasound-detected group and the ultrasound-missed group (*p* = 0.010, *p* = 0.011). Of the 11 tumors detected by ultrasound, four were extremely hypoechoic, two were hypoechoic, three were isoechoic, and two were hyperechoic. Color Doppler showed no blood flow in five tumors with the size range from 0.9 to 2.0 cm, and mild blood flow in six tumors with the size range from 2.8 to 5.9 cm.

**Conclusions:**

JGCT is rare, and has characteristic clinical manifestations. Diagnosis should be suspected in case of secondary hypertension, particularly in young women, if no renal vascular cause was found. Ultrasound, combined with clinical manifestations, was helpful for the diagnosis.

## Introduction

JGCT is a rare and benign renal tumor that originate from the juxtaglomerular cells of the capillary adventitia in the juxtaglomerular complex. The tumor is also called reninoma because it can secrete renin [1, 2]. Since the first report of JGCT in 1967 [3], fewer than 200 cases have been reported in the literature [4]. It is more common in young females aged 20–39 years, with a male: female ratio of 1:2 [5]. Occasionally cases in children and elderly patients were reported [2, 6, 7]. Typical clinical manifestations are hypertension, high renin activity, secondary aldosteronism, and hypokalemia [8], but some patients may have atypical clinical manifestations. Surgery is an effective treatment for JGCT [9–11].

Improvements in imaging techniques have led to a shorter interval between the occurrence of hypertension and the diagnosis of JGCT [12]. However, the diagnosis of JGCT remains challenging because it is rare, generally small, located in the renal cortex or subcortical regions, and may be missed or misdiagnosed [5]. Ultrasound could be helpful, however, the ultrasound manifestations of JGCT have not been fully studied. This study retrospectively summarized the clinical, laboratory, ultrasound and computed tomography (CT) characteristics of 15 JGCT patients and reviewed the literature to study the value of ultrasound in the diagnosis of JGCT.

## Methods

### Patients

This study was approved by the institutional review board of our hospital. From January 2005 to July 2020, fifteen patients diagnosed as JGCT by surgical pathology in Peking Union Medical College Hospital were collected. The clinical, laboratory, ultrasound, CT, surgical, and pathological features of the patients were analyzed retrospectively.

### Ultrasound examination

The ultrasound examination was performed by the PHLIPS HDI 5000, PHILIPS IU 22 machine (Philips Healthcare, Amsterdam, Netherland) and GE LOGIQ 9 (GE Healthcare, Wauwatosa, WI), equipped with 3.5–5 MHz probe, kidney presets. The grayscale and Color Doppler images of each patient were acquired and saved by radiologists with more than 5 years of experience in ultrasound examination. Image analysis was carried out by two radiologists with more than 5 years of experience with no knowledge of any clinical information. In case of disagreement between the two radiologists, the consensus was reached by discussion.

Image analysis included the location of the tumor (right/left kidney, cortex/cortex and medulla, protruded outward or not), size (the longest diameter), echo pattern (hypoechoic/isoechoic/ hyperechoic), shape (regular/irregular), margin (clear/indistinct), capsule (existent/ inexistent), blood flow signal (no/mild/abundant). Compared with the adjacent renal cortex, the echo pattern is divided into hypoechoic, isoechoic, and hyperechoic. The shape is classified as regular (round or lobulated), and irregular [13]. Clear margin meant that the tumor had a clear demarcation with the surrounding tissues. Otherwise, the margin was defined as indistinct. The capsule referred to the fibrous connective tissue membrane around the tumor, which appeared as a hyperechoic line on ultrasound. The blood flow was classified as no, mild (1–2 blood flows), and abundant (≥ 3 blood flows) according to Adler’s grading [14].

### Surgery and histopathological examinations

Surgeries were performed after the completion of necessary examinations. The location and size of the tumors were recorded during surgeries. Histopathological results were considered as the golden standard.

### Literature review

The literature search was conducted in Pub Medline and Embase with the keyword string “juxtaglomerular cell tumor ultrasound”. The study inclusion criteria included the diagnosis of JGCT with ultrasound descriptions (at least size and echo). The exclusion criteria were review articles, irrelevant or duplicate papers, and papers without full text available.

### Statistical analysis

SPSS 20.0 software was used for statistical analysis. Shapiro-wilk test was used to determine the normality of the data. Independent-samples T test or Fisher’s exact test was used to compare the differences between the two groups. P-values < 0.05 were considered statistically significant.

## Results

### Baseline characteristics

The clinical, ultrasound, and CT manifestations of 15 patients with JGCT were shown in Table 1. Among the 15 patients, there were 5 males and 10 females with a median age of 29 years (min-max, 10–72 years). Eleven (73.3%, 11/15) patients had grade III hypertension, three (20.0%, 3/15) patients had grade II hypertension, and one (6.7%, 1/15) patient had normal blood pressure. Six patients had blurred vision, six had dizziness or headache, and one had cerebral hemorrhage. The median interval from the diagnosis of hypertension to JGCT was 27 months (min-max, 1 month-12 years). Serum potassium was low in 10 patients (median 2.75 mmol/L, min-max, 2.3–3.2 mmol/L), and normal in 5 patients. The renin activity test was conducted in nine patients, and the results all showed high activity (median 6.17 ng/ml/h, min-max, 3.07–17.6 ng/ml/h). Plasma renin was tested in three patients, two of whom showed high levels (859 µIU/ml, 583 pg/ml) and one normal. The aldosterone test was performed in eleven patients, yielding ten elevated results (median: 348.95 pg/ml, min-max, 164.7–967 pg/ml) and one normal. Three patients were hospitalized for renal tumor surgery with no blood tests for reninoma, two of them had hypertension, and one had normal blood pressure. The serum potassium was normal in these three patients.

**Table 1:** Clinical, ultrasound, and CT manifestations of 15 patients with JGCT

No.	Year of diagnosis	Sex	Age	Clinical manifestations	Course of disease (m)	Disease site	Tu-mor size, cm	Ultrasound					CT	
								Echo	Shape	Margin	Capsule	Blood flow	Non-contrast enhanced CT	Contrast-enhanced CT(Parenchymal phase)
1	2020	Female	27	Hypertension, high renin, high aldosterone, low potassium Diplopia, polydipsia, polyuria	7	Left /cortex and medulla	1.5	Missed	**-**	**-**	-	-	Slightly high density	Moderate
2	2019	Female	29	Asymptomatic	1	Left /cortex/ protrusion outward	3.7	Hyperechoic	Regular	Clear	Inexistent	Mild	Mixed	Mild
3	2019	Female	29	Hypertension, high renin, high aldosterone, low potassium Dizziness, headache, blurred vision	48	Right /cortex	0.9	Isoechoic	Regular	Indistinct	Inexistent	No	Low density	Mild
4	2018	Female	29	Hypertension, high renin, high aldosterone, low potassium Dizziness, blurred vision	18	Left/ cortex and medulla	1.3	Isoechoic	Regular	Clear	Inexistent	No	Isodensity	Mild
5	2012	Male	40	Hypertension, increased renin activity	144	Right/cortex/ protrusion outward	1.5	Isoechoic	Regular	Clear	Inexistent	No	Slightly high density	Mild
6	2012	Female	29	Hypertension, increased renin activity, low potassium Fatigue, retinopathy	6	Left/cortex	1.2	Missed	**-**	**-**	**-**	**-**	Missed	Mild
7	2012	Male	10	Hypertension, high renin, high aldosterone, low potassium Headache, cerebral hemorrhage, diminution of vision	24	Right/cortex	1.2	Missed	**-**	**-**	**-**	**-**	Slightly High density	Mild
8	2011	Male	46	Hypertension	72	Left/cortex and medulla/ protrusion outward	2.0	Extremely hypoechoic	Regular	Indistinct	Inexistent	No	Mixed	Mild
9	2010	Female	37	Hypertension, high renin, high aldosterone, low potassium Dizziness, nausea, vomiting	84	Left/cortex and medulla/ protrusion outward	5.9	Extremely hypoechoic	Irregular	Indistinct	Inexistent	Mild	Low density	Moderate
10	2009	Female	18	Hypertension, high renin, high aldosterone, low potassium	1	Right/cortex and medulla/ protrusion outward	3.8	Hypoechoic	Regular	Clear	Existent	Mild	Slightly Low density	Mild
11	2008	Female	72	Hypertension	36	Left/cortex and medulla/ protrusion outward	3.0	Extremely hypoechoic	Regular	Clear	Inexistent	Mild	Low density	Moderate
12	2008	Male	16	Hypertension, high renin, high aldosterone, low potassium Headache, dizziness, fatigue	1	Right/cortex/ protrusion outward	3.0	Hypoechoic	Regular	Clear	Inexistent	Mild	Low density	Mild
13	2007	Male	39	Hypertension, high renin, high aldosterone, low potassium	132	Right/cortex/ protrusion outward	1.2	Extremely hypoechoic	Regular	Clear	Existent	No	Isodensity	Mild
14	2006	Female	15	Hypertension, high renin, high aldosterone, low potassium Headache, blurred vision, fundus hemorrhage	12	Right/cortex	1.0	Missed	**-**	**-**	**-**	**-**	Missed	Mild
15	2005	Female	26	Hypertension	4	Left/cortex and medulla/ protrusion outward	2.8	Hyperechoic	Regular	Clear	Inexistent	Mild	Isodensity	Mild

All 15 JGCTs were solitary tumors, including seven in the right kidney and eight in the left kidney. The median longest diameter of the tumors was 1.5 cm (min-max, 0.9–5.9 cm).

### Ultrasound manifestations

Among the 15 JGCTs, 11 were correctly detected by preoperative ultrasound, and 4 were missed.

**Fig. 1:**
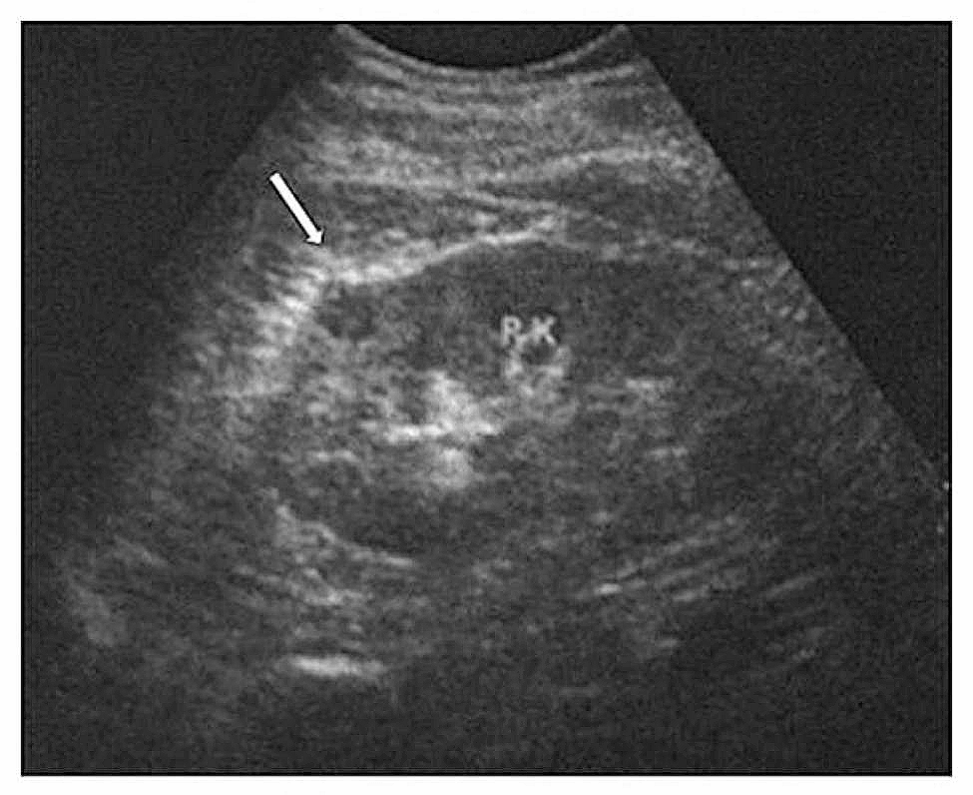
A JGCT lesion in the right kidney. Ultrasound showed extremely hypoechoic lesion (arrow), 1.2 × 1.0 × 0.8 cm,with regular morphology and clear boundary

**Fig. 2:**
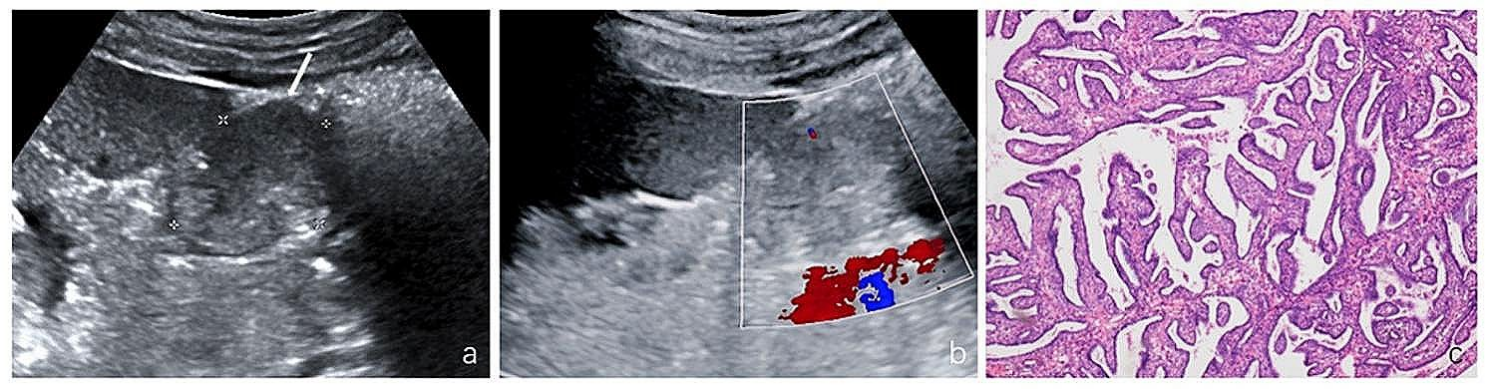
A JGCT lesion in the left kidney. **(a)** Grayscale ultrasound showed isoechoic lesion (arrow), 4.0 × 3.4 × 3.1 cm, with regular morphology and clear boundary. **(b)** Color Doppler ultrasound showed mild blood flow. **(c)** Morphologic features on hematoxylin-eosin stain. The diagnosis of JGCT was confirmed by the postoperative pathology

Of the 11 tumors detected by ultrasound, the median longest diameter of the tumors was 2.8 cm (min-max, 0.9–5.9 cm). Nine protruded outward, one protruded toward the collecting system, and one was completely in the renal cortex with no protrusion. Four were extremely hypoechoic (Fig. 1), two were hypoechoic, three were isoechoic (Fig. 2), and two were hyperechoic. The morphology was regular in ten cases and irregular in one case. The margin was clear in eight cases and indistinct in three cases. Ultrasound showed a capsule in two cases and no capsule in nine cases. Color Doppler showed no blood flow in five tumors with the size range from 0.9 to 2.0 cm, and mild blood flow in six tumors with the size range from 2.8 to 5.9 cm. The preoperative diagnosis was one reninoma, one renal cell carcinoma, and nine undetermined tumors.

Of the 4 tumors missed by ultrasound, the longest diameter was 1.0 cm, 1.2 cm, 1.5 cm, and 1.2 cm respectively. All of them located in the renal cortex with no protrusion outward.

There was a significant difference in tumor size (2.64 ± 1.48 cm vs. 1.23 ± 0.21 cm, Independent-samples T test, *p* = 0.010) between the ultrasound-detected group and ultrasound-missed group. There was also a significant difference in whether the tumor protruded outward (9/11 vs. 0/4, Fisher’s exact test, *p* = 0.011) between the two groups (Table 2).

**Table 2:** Comparison of whether the tumor protruded outward between the ultrasound-detected group and ultrasound-missed group

	Protrusion outward or not		
Ultrasound	Protrusion	No Protrusion	Total
Detected	9	2	11
Missed	0	4	4
Total	9	6	15

### CT manifestations

Of the 11 tumors detected by ultrasound, non-contrast enhanced CT showed five low density, three isodensity, one slightly high density and two mixed density. Dynamic contrast-enhanced CT showed nine mild enhancement, and two moderate enhancement in parenchymal phase. The preoperative CT diagnosis was three reninoma, one renal cell carcinoma, and seven undetermined tumors.

Of the 4 lesions missed by ultrasound, two tumors located in the cortex, with the size of 1.0 and 1.2 cm respectively, showed similar density with the cortex on non-contrast enhanced CT and mild enhancement during parenchymal phase on contrast enhanced CT. One tumor located in the cortex with the size of 1.2 cm, showed slightly high density on non-contrast enhanced CT and mild enhancement during parenchymal phase on contrast enhanced CT. One tumor located in the cortex and medulla, with the size of 1.5 cm, showed slightly high density on non-contrast enhanced CT and moderate enhancement during parenchymal phase on contrast enhanced CT. The preoperative CT diagnosis was three reninoma, and one renal cell carcinoma.

### Surgery and follow-up

All 15 patients underwent partial nephrectomy. Two of them underwent intraoperative ultrasound localization, which successfully guided the laparoscopic partial nephrectomy. The blood pressure returned to normal in 13 patients after operation. No tumor relapse was found in any of the 15 patients during the follow-up of 6 months to 15 years.

### Literature review

Eighty-five papers (65 papers in PubMed and 20 papers in Embase) were retrieved. A total of 25 patients in 13 papers met the inclusion criteria including 17 females and 8 males with a median age of 24 years (11–57 years) [4, 11, 15–25]. All tumors were solitary, with a median size of 3.3 cm (0.6–6.5 cm). Two JGCTs were extremely hypoechoic, 12 were hypoechoic, 1 were isoechoic, 10 were hyperechoic.

## Discussion

JGCT is a rare renal tumor. Haab et al. reported eight cases of JGCT among 30,000 hypertensive patients in a 15-year period [26]. The tumor is usually solitary, small in size (< 3 cm, rarely > 4 cm) [1, 5]. Surgery is an effective treatment. To our knowledge, this is the largest report describing the clinical, laboratory, ultrasound and CT manifestations of JGCT, providing detailed descriptions of the ultrasound characteristics of 15 cases.

In our series, JGCT showed extremely hypoechoic, hypoechoic, isoechoic or hyperechoic, with no or mild blood flow, as described previously [4–8, 10, 11, 19, 20, 22, 23, 25–29]. Hypoechoic, especially extremely hypoechoic, may be explained by the closely packed cellular, highly organized, and compact architectural organization, as seen in other endocrine tumors. Extremely hypoechoic might be the characteristics, however, the data remained insufficient and further study is needed. The tumor showed no or mild blood flow might be related to the vasoconstriction caused by renin and the decreased blood flow caused by the proliferation of intima and middle layer of tumor arterioles [29]. Pathologically, JGCT generally has fibrous capsule [11, 22, 28], however we observed capsule in only two patients, which might be due to the thin capsule hardly detected by ultrasound.

The ultrasound diagnosis of JGCT is still challenging. In our study, four tumors, with small size (1.0∼1.5 cm) and located completely in the cortex with no protrusion, were missed by ultrasound. Two of them showed similar density with the cortex and were missed on the non-contrast enhanced CT. Because ultrasound and non-enhanced CT can fail, contrast enhanced CT should be performed when a JGCT is suspected. The contrast enhanced CT was positive in all of the 15 JGCTs in our study, as well as in the cases reported [5, 26, 30–32]. Taken together, contrast enhanced CT should be considered in all cases of JGCT. Contrast enhanced ultrasound might be helpful [4, 25]. Li et al. reported two cases of JGCT, invisible by previous conventional ultrasound, found by contrast enhanced ultrasound. They were complete endophytic, and showed hypoenhancement, slow wash in and slow wash out. One was 1.0 × 1.0 × 0.7 cm, the other was 3.0 × 2.0 × 1.0 cm [25]. The small sample size precluded a firm recommendation of contrast enhanced ultrasound for JGCT diagnosis. JGCT may have specific features in enhanced ultrasound, which needs to be confirmed by future studies. However, the application of enhanced ultrasound needs to be based on the two-dimensional ultrasound localization of the lesion. Therefore, enhanced ultrasound may not be helpful in reducing ultrasound misdiagnosis of JGCT cell tumors, which requires other solutions.

The limitation of our study was the small number of cases and techniques such as contrast enhanced ultrasound was not applied. Most of the studies were case reports, so we did not conduct a meta-analysis. A multicenter study and more in-depth prospective studies should be conducted in the future to provide a more comprehensive information of this rare tumor.

## Conclusions

JGCT is rare, and has characteristic clinical manifestations. Diagnosis should be suspected in case of secondary hypertension, particularly in young women, if no renal vascular cause is found. Ultrasound, combined with clinical manifestations, was helpful for the diagnosis.

## Data Availability

The data and material can be provided if asked on a basis of good reasons.

## Abbreviations

JGCT: juxtaglomerular cell tumor
CT: computed tomography

## References

[1] Duan X, Bruneval P, Hammadeh R, Fresco R, Eble JN, Clark JI, Vigneswaran WT, Flanigan RC (2004). Picken MM.Metastatic juxtaglomerular cell tumor in a 52-year-old man. Am J Surg Pathol.

[2] Shera AH, Baba AA, Bakshi IH, Lone IA (2011). Recurrent malignant juxtaglomerular cell tumor: a rare cause of malignant hypertension in a child. J Indian Assoc Pediatr Surg.

[3] Robertson PW, Klidjian A, Harding LK, Walters G, Lee MR (1967). Robb-Smith AHT.Hypertension due to a renin-secreting renal tumour. Am J Med.

[4] Zhang R, Xu M, Xie XY (2021). The role of Real-Time contrast-enhanced Ultrasound in Guiding Radiofrequency ablation of Reninoma: Case Report and Literature Review. Front Oncol.

[5] Wong L, Hsu THS, Perlroth MG, Hofmann LV, Haynes CM, Katznelson L. Reninoma: case report and literature review. J Hypertens 2008, 26. 10.1097/HJH.0b013e3282f283f3 18192852

[6] Onder S, Baydar DE (2011). A case of juxtaglomerular cell tumor with novel histologic features. Int J Surg Pathol.

[7] Shao L, Manalang M, Cooley L (2008). Juxtaglomerular cell tumor in an 8-year-old girl. Pediatr Blood Cancer.

[8] Elouazzani H, Jahid A, Bernoussi Z (2014). Mahassini N.Juxtaglomerular cell tumor: a distinct mesenchymal tumor of kidney. J Clin Imaging Sci.

[9] Jiang S, Yang Y, Wu R, Yang Q, Zhang C, Tang Y, Mo C (2020). Characterization and management of Juxtaglomerular Cell Tumor: analysis of 9 cases and literature review. Balkan Med J.

[10] Mezoued M, Habouchi MA, Azzoug S, Mokkedem K, Meskine D, JUXTAGLOMERULAR CELL CAUSE OF SECONDARY HYPERTENSION IN AN ADOLESCENT (2020). Acta Endocrinol (Buchar).

[11] Trnka P, Orellana L, Walsh M, Pool L, Borzi P (2014). Reninoma: an uncommon cause of renin-mediated hypertension. Front Pediatr.

[12] Ruyer A, Millet I, Ribstein J, Taourel P (2011). [Renin-producing tumor: a rare case of curable hypertension. A case report]. J Radiol.

[13] <number>13.</number>ACR BI-RADS breast imaging and reporting data system (2013). Breast imaging atlas.

[14] Adler DD, Carson PL, Rubin JM, Quinn-Reid D (1990). Doppler ultrasound color flow imaging in the study of breast cancer: preliminary findings. Ultrasound Med Biol.

[15] Kashiwabara H, Inaba M, Itabashi A, Ishii J, Katayama SA (1997). Case of renin-producing Juxtaglomerular Tumor: effect of ACE inhibitor or angiotensin II receptor antagonist. Blood Press.

[16] Yang H, Wang Z, Ji J (2016). Juxtaglomerular cell tumor: a case report. Oncol Lett.

[17] Chambô JL, Falci Júnior R, Lucon AM (2004). Juxtaglomerular cell tumor as a rare cause of hypertension in adults. Int Braz J Urol.

[18] Squires JP, Ulbright TM, DeSchryver-Kecskemeti K (1984). Engleman W.Juxtaglomerular cell tumor of the kidney. Cancer.

[19] Lachvac L, Svajdler M, Valansky L, Nagy V, Benicky M, Frohlichova L (2011). Nyitrayova O.Juxtaglomerular cell tumor, causing fetal demise. Int Urol Nephrol.

[20] Tessi C, Szklarz MT, Vásquez M, López Imizcoz F, Ruiz J, Weller S, Villoldo G, Sager C, Burek CM (2020). Corbetta JP.Laparoscopic Nephro-Sparing surgery of a Reninoma Tumor in a Pediatric patient. Urology.

[21] Xue M, Chen Y, Zhang J, Guan Y, Yang L, Wu B (2017). Reninoma coexisting with adrenal adenoma during pregnancy: a case report. Oncol Lett.

[22] Dunnick NR, Hartman DS, Ford KK, Davis CJ, Amis ES (1983). Jr.The radiology of juxtaglomerular tumors. Radiology.

[23] Chen Z, Tang Z-Y, Liu H-T, Chen X (2014). Treatment of Juxtaglomerular Cell Tumor of the kidney by retroperitoneal laparoscopic partial nephrectomy. Urol J.

[24] Weiss JP, Pollack HM, McCormick JF, Malloy TM, Hanno PM, Carpiniello VL (1984). Renal hemangiopericytoma: surgical, radiological and pathological implications. J Urol.

[25] Qiuyang Li M, Ying Zhang MD, Yong Song MD, Aitao Guo MD, Nan Li BS, Yukun Luo MD (2020). Jie Tang, MD.Clinical Application of Ultrasound in the diagnosis and treatment of Reninoma. Adv Ultrasound Diagnosis Therapy.

[26] Haab F, Duclos JM, Guyenne T, Plouin PF, Corvol PR (1995). Secreting tumors: diagnosis, Conservative Surgical Approach and Long-Term results. J Urol.

[27] Brandal P, Busund LT, Heim S (2005). Chromosome abnormalities in juxtaglomerular cell tumors. Cancer.

[28] Hagiya A, Zhou M, Hung A, Aron MJ (2020). Cell Tumor with atypical pathological features: report of a case and review of literature. Int J Surg Pathol.

[29] Prasad SR, Surabhi VR, Menias CO, Raut AA, Chintapalli KN (2008). Benign renal neoplasms in adults: cross-sectional imaging findings. AJR Am J Roentgenol.

[30] Corvol P, Pinet F, Plouin PF, Bruneval P, Menard J (1994). Renin-secreting tumors. Endocrinol Metab Clin North Am.

[31] Tanabe A, Naruse M, Ogawa T, Ito F, Takagi S, Takano K, Ohashi H, Tsuchiya K, Sone M, Nihei H (2001). Dynamic computer tomography is useful in the Differential diagnosis of Juxtaglomerular Cell Tumor and Renal Cell Carcinoma. Hypertens Res.

[32] Faucon A-L, Bourillon C, Grataloup C, Baron S, Bernadet-Monrozies P, Vidal-Petiot E, Azizi M, Amar L (2019). Usefulness of Magnetic Resonance Imaging in the diagnosis of Juxtaglomerular Cell tumors: a report of 10 cases and review of the literature. Am J Kidney Dis.

